# Indirect costs associated with skin infectious disease in children: a systematic review

**DOI:** 10.1186/s12913-021-07189-3

**Published:** 2021-12-11

**Authors:** Irene Lizano-Díez, Jesús Naharro, Ilonka Zsolt

**Affiliations:** Ferrer Internacional, S.A., Av Diagonal 549, 5th floor, 08029 Barcelona, Spain

**Keywords:** Skin infectious diseases, Cost of illness, Indirect costs, Child, SSTIs, Systematic review

## Abstract

**Background:**

There are limited data in the literature on the indirect costs associated with skin and soft tissue infections (SSTIs) in the pediatric population. This study aimed to conduct a systematic review of the indirect costs associated with SSTIs in children.

**Methods:**

The search was conducted in PubMed, SCOPUS, and Web of Science up to January 2020. Thirteen search strategies were designed combining MeSH terms and free terms. SSTIs were defined as bacterial or viral infections, dermatomycoses, and parasitic infestations. Only primary studies were included. All analyzed costs were converted to 2020 Euros.

**Results:**

Thirteen of the identified publications presented indirect costs of SSTIs in children and were conducted in Argentina, Australia, Brazil, Hungary, New Zealand, Poland, Spain, Taiwan, and the USA. Nine studies described indirect costs associated with infection of *Varicella-zoster virus*: lost workdays by outpatient caregivers ranged from 0.27 to 7.8, and up to 6.14 if caring for inpatients; total productivity losses ranged from €1.16 to €257.46 per patient. Three studies reported indirect costs associated with acute bacterial SSTIs (community-associated methicillin-resistant *Staphylococcus aureus*) in children: total productivity losses ranged from €1,814.39 to €8,224.06 per patient, based on impetigo, cellulitis, and folliculitis. One study of parasitic infestations (*Pediculus humanus capitis*) reported total indirect costs per patient of €68.57 (formal care) plus €21.41 due to time lost by parents in purchasing treatment.

**Conclusions:**

The economic burden of SSTIs is highly relevant but underestimated due to the lack of studies reporting indirect costs. Further cost studies will allow a better understanding of the magnitude of the financial burden of the disease.

**Supplementary Information:**

The online version contains supplementary material available at 10.1186/s12913-021-07189-3.

## Background

The global impact of skin diseases, both social and economic, is significant, with 4.86 billion incident cases of skin and subcutaneous diseases estimated worldwide in 2019 [1].

Though taxonomies may vary, skin and soft-tissue infections (SSTIs) as a category include a broad set of pathological conditions that involve the skin and its underlying structure and range from uncomplicated superficial infections to more severe infections that extend to the subcutaneous tissue, fascia, or muscle [2, 3]. Skin infections are a frequent occurrence in the pediatric population and are generally more severe than in adolescents and adults, a difference that is due in large part to the functional impairment of immune defenses during the first period of life, as evidenced in both the innate and adaptive response [4].

Many SSTIs are caused by bacteria and are referred to as *acute bacterial skin and skin structure infections* (ABSSSIs). These infections can pose a challenge for the medical community, particularly with the increase in antimicrobial resistance, and are associated with high direct and indirect costs to both the healthcare system and society. Antimicrobial resistance is a growing concern and the increase in the incidence of infections caused by bacteria that have developed resistance to previously effective antimicrobials, such as methicillin-resistant *Staphylococcus aureus* (MRSA), has led to higher rates of complications, hospitalizations and, occasionally even death [5, 6]. The prevalence of community-acquired MRSA (CA-MRSA), meaning MRSA colonization and infection that is not associated with a healthcare setting, among children with no identified risk factors is also on the rise and is becoming a public health problem [7, 8].

Other SSTIs are caused by viruses, most notably the *Varicella-zoster virus* (VZV), which is the causal agent of chickenpox and shingles [9]. Despite the ubiquity of this virus, there is still much to learn about the epidemiology and burden of varicella, particularly in Europe, given that the majority of studies took place in a hospital setting. Therefore, although there has been an increase in the data on non-hospitalized patients in recent years, the data are still limited or underestimated [10].

Parasitic infestations are common among children and adolescents. The head louse (*Pediculus humanus capitis*) is an obligate human ectoparasite that causes head lice infestation (*pediculosis capitis*) and is estimated to affect more than 100 million people worldwide every year [11]. The infestation, which affects the hair and scalp [12], is not associated with systemic disease; however, it can lead to anxiety, embarrassment, work and/or school absenteeism, productivity loss and significant medical costs [13–15].

SSTIs impose a burden on patients, their families and society that is multidimensional: the impact is psychological, social and financial. The negative impact of SSTIs may be greater for children due to their fragility and resource needs and their quality of life (QoL) can be deeply affected by these infections. Among the indirect costs associated with childhood diseases are time taken off from work by parents or other caregivers when a child must stay home from school due to illness, time spent traveling to and from hospitals and other healthcare facilities, and the time spent at the healthcare facility while the child receives treatment. Quantifying the burden for a child affected by the SSTI, however, is difficult and has not been estimated. It would need to account, for example, for lost activity, the inability to perform certain tasks, and the cost of missed educational opportunities due to school absenteeism.

There are many people worldwide who are vulnerable to the economic consequences of illness, the costs of which can be either direct or indirect. The indirect cost of disease is an important factor and should be taken into account when measuring the additional impact of diseases beyond the traditional direct medical costs. Though these indirect costs are difficult to quantify due to the lack of quality data, they could represent a significant fraction of the total cost associated with many diseases. Indirect costs are the loss of earnings resulting from an adverse health outcome. Decreased productivity can arise from three situations: absence from paid work (absenteeism), including sick leave, early retirement, premature death and reduced employment (or unemployment); reduced productivity during paid work (presenteeism); and changes in unpaid work [16]. In the case of pediatric patients, productivity loss is mainly incurred by their caregivers. Each category of indirect costs can be calculated using either the human capital approach or the friction cost approach. The human capital approach converts the gross income that will not be obtained in the future due to disease into real costs from a social perspective. The friction cost approach takes into account productivity losses until a substitute is hired to replace the sick worker [16].

There are limited data in the literature on the indirect costs associated with SSTIs in the pediatric population. Therefore, we aimed to conduct a systematic literature review to collect and summarize current data on the indirect costs associated with SSTIs in children worldwide.

## Methods

We carried out a systematic review to retrieve studies reporting the indirect costs of SSTIs in the pediatric population. This study followed the guidelines for conducting systematic reviews of economic evaluations studies published by the Centre for Reviews and Dissemination – CRD [17] and the research protocol was registered in Open Science Framework (https://osf.io/wrvap/). The methods and findings of this systematic review were reported following the Preferred Reporting Items for Systematic Reviews and Meta-Analysis (PRISMA) guidelines [18]. The PRISMA checklist is available in Online Resource 1.

A PICO approach, the details of which are available in our study protocol, was used to formulate the following research question: What is the estimation of indirect costs in managing SSTIs in the pediatric population aged 1 to 12 years? [19]. The databases searched were PubMed, SCOPUS and Web of Science (WoS) (up to January 2020). To identify relevant studies, we designed search algorithms that combined controlled terms from each database with free terms, such as: “skin diseases, infectious”, “child”, “cost of illness”, “soft tissue infections”, “indirect costs”, “staphylococcal skin infections”, and “human”. A complete description of the search strategies can be found in Online Resource 2. In addition, the references cited in the literature reviews and studies retrieved were also checked to identify other relevant studies not found using our search strategy. No date, language or geographic limits were applied.

**Fig. 1:**
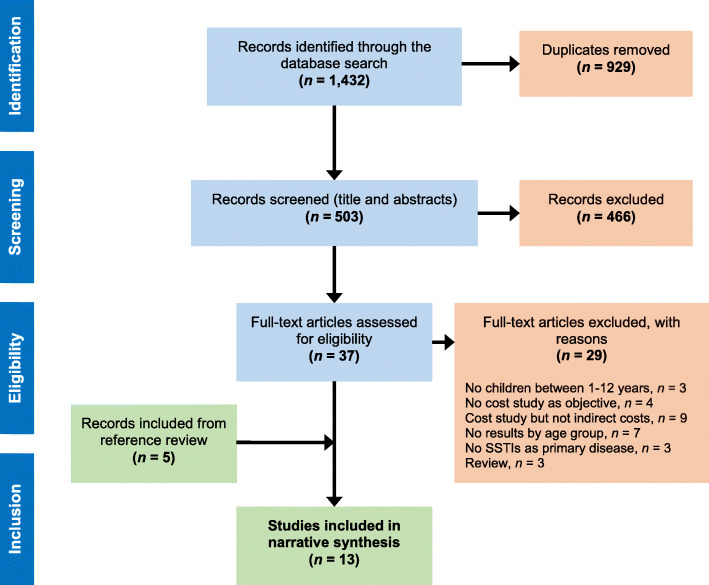
PRISMA flow chart for the global search with regional-level outcomes. The search strategies returned 1432 records, 929 of which were duplicates. After screening the titles and abstracts of the remaining 503 references, a further 466 were excluded. The full texts of 37 articles were assessed for eligibility, of which 13 were found eligible and included in the analysis. Of the full-text articles assessed, 29 were excluded for the following reasons: No children between 1 and 12 years (3); No cost study as objective (4); Cost study but not indirect costs (9); No results by age group (7); No SSTIs as primary disease (3); and Review (3)

We included primary studies reporting health economic analyses of the impact of SSTIs (defined as bacterial or viral infections, dermatomycoses and parasitic infestations) in the pediatric population from 1 to 12 years of age. Studies with a broader age range were included so long as there was overlap with our specified age range (1 to 12). We included studies that measured indirect costs, defined as lost earnings and productivity of both patients and their informal caregivers, and excluded studies that reported only direct costs as well as those focused on the diagnosis, treatment, epidemiology, or non-economic aspects of SSTIs. The complete list of reasons for the exclusion of articles is included in Fig. 1.

The references identified from the initial search were screened using the criteria described above to exclude irrelevant studies based on title and abstract. The full texts of the remaining articles were independently reviewed by two researchers, following the selection criteria described earlier, and disagreements regarding inclusion or exclusion of studies were resolved by consensus.

For each study we carried out a quality appraisal using an adapted version of a previously developed critical appraisal checklist for cost-of-illness (COI) studies [20]. Each potentially eligible study was evaluated for relevance, methodological robustness, and reporting. The results of the quality appraisal are available in Online Resource 3. We used a predefined data extraction form to collect the relevant data from the selected studies. One reviewer evaluated the studies and extracted the data, while another reviewer checked the accuracy and completeness. The following data were extracted from each study: year and journal of publication, first author, country of the study, study design, skin disease, pathogen, sample size, age, and data on indirect cost (workdays lost by caregivers, school days lost, total productivity losses, annual indirect cost, and formal childcare costs). The primary outcome was any available data on indirect costs related to SSTIs in children. Extracted costs were converted to 2020 Euros by first inflating the original currency and then converting it to Euros using the web-based tool provided by the Campbell and Cochrane Economics Methods Group (CCEMG-EPPI) [21] along with the Organization for Economic Cooperation and Development (OECD) purchasing power parity index and the inflation index of Euro area countries [22]. For non-economic studies, the risk of bias was assessed using the National Institute for Health and Care Excellence (NICE) scale to assess the risk of bias in observational studies [23] and for model-based economic evaluations, the Bias in Economic Evaluation (ECOBIAS) checklist [24] was used (for details, see Online Resource 4 and Online Resource 5, respectively). We conducted a qualitative narrative synthesis, and the study characteristics were summarized in figures and summary tables.

## Results

The search yielded 503 unique references, of which 466 were excluded through title-and-abstract screening; 37 articles were reviewed in full text. Twenty-nine articles were excluded based on full-text evaluation, and 5 articles identified from a review of references were added, bringing the final total to 13 studies. The complete eligibility process is described in a PRISMA flow chart in Fig. 1. Five of the identified studies were economic evaluations [8, 14, 25–27], 1 was an economic analysis in the context of a head-to-head randomized clinical trial [28] and 7 were retrospective chart/case reviews [6, 10, 28–32], 3 of which were multicenter [30, 32, 33]. We assessed the risk of bias for all studies. Most had a moderate risk of bias (between 57.1 and 88.2% of criteria fulfilled; see Online Resources 4 and 5).

The studies were carried out in 9 different countries and published between 1992 and 2018. Five were conducted in the United States [6, 8, 14, 28, 31] and 1 study was published from each of the following countries: Argentina [30], Australia [29], Brazil [26], Hungary [33], New Zealand [27], Poland [32], Spain [10], and Taiwan [25]. The key characteristics of the studies are summarized in Table 1 and detailed indirect costs and variables are summarized in Table 2. In most of the studies (12 studies, 92%), indirect costs accounting for productivity losses were estimated using the human capital approach. No information regarding the method of indirect cost calculation was reported for the remaining study [6]. No studies of dermatomycoses that fulfilled inclusion criteria were retrieved for full-text analysis.

**Table 1:** Summary of primary studies on indirect costs of SSTIs in the pediatric population

Reference	Country	Study Design	Skin Disease (Group)	Pathogen	Sample Size	Age	Indirect costs and related variables collected
Lee et al. (2013) [8]	USA	Economic simulation model	Impetigo, cellulitis, and folliculitis (Bacterial infection)	CA-MRSA	NA	0–15 years	Total productivity loss, including absenteeism and mortality, associated with CA-MRSA
Rice et al. (1992) [28]	USA	Cost-effectiveness analysis based on head-to-head randomized clinical trial	Impetigo (Bacterial infection)	*Staphylococcus aureus* *Group A Streptococcus*	93	3 months-16 years	Average cost of school days; average wage of workdays. *For Erythromycin or Mupirocin treatment* Number of school days lost; number of workdays lost; total cost of school days lost; and total cost of workdays lost.
Wright et al. (2013) [6]	USA	Retrospective comparative case review (cost-effectiveness analysis)	Subcutaneous abscesses (Bacterial infection)	*Staphylococcus aureus*	344	< 18 years	*Traditional packing and Minimally invasive treatment:* Number of workdays lost; total hospital indirect cost.
Díez-Domingo et al. (2003) [10]	Spain	Retrospective chart review	Chickenpox (Viral infection)	*Varicella-zoster virus*	683	0–14 years	Average school days lost; average workdays lost; unit cost by day off work; average cost for productivity loss; and babysitter cost.
Ferson et al. (1998) [29]	Australia	Retrospective chart review	Chickenpox (Viral infection)	*Varicella-zoster virus*	174	8 months-5 years	Number of workdays lost for mothers and fathers; average wage for parents; total cost for productivity loss for mothers and fathers; average cost of childcare by a nanny.
Giglio et al. (2018) [30]	Argentina	Multi-center, retrospective chart review	Chickenpox (Viral infection)	*Varicella-zoster virus*	150	1–12 years	Number of workdays lost for outpatients and inpatients; total cost for productivity loss for outpatients and inpatients; and total annual indirect cost (all cases).
Hsu et al. (2003) [25]	Taiwan	Cost-benefit analysis	Chickenpox (Viral infection)	*Varicella-zoster virus*	NA	0–20 years	Number of workdays lost, cost unit per day off work; total cost for productivity loss, estimation of total indirect cost reduction for vaccination (all cases).
Lieu et al. (1994) [31]	USA	Retrospective chart review	Chickenpox (Viral infection)	*Varicella-zoster virus*	179	0–6 years	Number of workdays lost for mothers and fathers; average school days lost; total cost for productivity loss for mothers and fathers; average cost for productivity loss per child; and babysitter median cost.
Meszner et al. (2017) [33]	Hungary	Multi-center, retrospective chart review	Chickenpox (Viral infection)	*Varicella-zoster virus*	156	1–12 years	Number of workdays lost for outpatients and inpatients; total cost for productivity loss for outpatients and inpatients; and total annual indirect cost (all cases).
Scuffham et al. (1999) [27]	New Zealand	Cost-effectiveness analysis	Chickenpox (Viral infection)	*Varicella-zoster virus*	NA	< 19 years	Number of workdays lost for outpatients and inpatients; average wage for parents; total cost for productivity loss for outpatients and inpatients; and average cost-savings due to vaccination from avoided work-loss.
Valentim et al. (2008) [26]	Brazil	Cost-effectiveness analysis	Chickenpox (Viral infection)	*Varicella-zoster virus*	NA	0–15 years	Number of workdays lost for outpatients and inpatients; average wage for parents; and total cost for productivity loss for outpatients and inpatients.
Wysocki et al. (2018) [32]	Poland	Multi-center, retrospective chart review	Chickenpox (Viral infection)	*Varicella-zoster virus*	150	1–12 years	Number of workdays lost for outpatients and inpatients; total cost for productivity loss for outpatients and inpatients; and total annual indirect cost (all cases).
Gur et al. (2009) [14]	USA	Cost-effectiveness analysis	Head lice infestation (Parasitic infestation)	*Pediculus humanus capitis*	NA	3–12 years	Number of school days lost; time of children home care/formal care, time lost due to medical visits and shopping treatment; average wage for parents; average wage of formal care; total home care cost/formal care; and total cost for productivity loss.

*CA-MRSA* Community-associated methicillin-resistant *Staphylococcus aureus*

**Table 2:** Indirect costs of SSTI (Euro^a^)

First Author-Country and Date	Sample Size	Subgroup	Daily school cost, €^**a**^	Avg. daily wage per parent, €^**a**^	Avg. No. school days lost per patient	Avg. No. workdays lost per patient	Total cost school days lost, €^**a**^	Total cost productivity loss, €^**a**^	Total hospital indirect cost, €^**a**^	Total annual indirect cost (all cases), €^**a, b**^	Total indirect cost reduction for vaccination, €^**a**^	Avg. cost nanny per child and process, €^**a**^
**Bacterial infection**												
Lee-USA-2013 [8]	NA							1,814.39–8,224.06				
Rice-USA-1992 [28]	93		29.49	78.79/day								
		Erythromycin			3.0 ± 3.5	0.5 ± 0.8	79.46 ± 92.70	39.33 ± 59.74				
		Mupirocin			1.2 ± 1.7	0.2 ± 0.7	31.78 ± 45.03	14.93 ± 52.27				
Wright-USA-2013 [6]	344	–										
		TP				2 (IQR 1-2)			1,824.54^c^			
		MI				1 (IQR 1–1.25)			1,529.96^c^			
**Viral infection**												
Díez-Domingo- ESP-2003 [10]	683			84.55/day	6.22 ± 5.29	0.97 (0.5–15)		82.01 (42.27–1,268.23)				11.95
Ferson-AUS1998 [29]	174											19.61
		Mother		94.89/day		2.5 (95% CI:2.0–2.9)		234.34				
		Father		113.89/day		0.42 (95% CI:0.26–0.58)		47.83				
Giglio-ARG-2018 [30]	150									17,382,154.02		
		Outpatients				7.8		141.91				
		Inpatients				4.8		98.17				
Hsu-TWN-2003 [25]	NA			51.08/day		1.85		94.5			31.66 mill (all cases)	
Lieu-USA-1994 [31]	179							210.55 ^e^				52.12 (10.42-312.70) ^d^
		Mother				2.5		257.46				
		Father				0.8		174.07				
Meszner-HUN-2017 [33]	156									4,308,232.09		
		Outpatients				2.5		114.21				
		Inpatients				3.6		191.76				
Scuffham-NZL-1999 [27]	NA			38.61/day							70.60 (per vaccinated child)	
		Outpatients				3		115.84				
		Inpatients				6.14		237.08				
Valentim-BRA-2008 [26]]	156											
		Outpatients				0.27		1.16				
		Inpatients				4.78–4.84		20.61–20.87				
Wysocki-POL-2018 [32]	150									34,623,835.89		
		Outpatients				2.7		201.03				
		Inpatients				4.7		251.71				
**Parasitic infestation**												
Gur USA-2009 [14]	NA			21.41/h	1	0.04		21.41				68.57

^a^Cost in Euros adjusted to 2020 Euros. ^b^Based in the annual number of cases of VZV infection for 2015: *n =* 133,434 [29]; *n =* 172,117 [31]; *n* = 37,585 [32]. ^c^Significant differences between traditional packing and minimally invasive treatment (*p* < 0.001). ^d^ Value corresponds to median.*;* ^e^ Average cost for productivity loss per child. *Avg* Average, *CI* Confidence interval, *IQR* Interquartile range, *MI* Minimally invasive treatment, *NA* Not applicable, *No* Numbers, *TP* Traditional packing treatment. Total annual indirect cost considers the total number of infections in the season and includes indirect costs derived from productivity loss. Total cost productivity loss refers to costs due to work absence or loss of productivity

### Varicella-zoster virus

Of the studies in this review, the majority were on chickenpox, with a total of 9 studies reporting indirect costs associated with VZV infection [10, 25–27, 29–33]. The number of reported workdays missed ranged from 0.27 to 7.8 days for caregivers of outpatients, and up to 6.14 days for those caring for inpatients. For non-complicated infection, the cost of lost productivity ranged from €1.16 (Brazil) [26] to €257.46 (United States) [31]. In the case of carers of inpatients, the costs of productivity loss ranged from €20.61 (Brazil) [26] to €251.71 (Poland) [32]. Two studies reported that productivity loss was higher for working mothers than fathers. Ferson et al. and Lieu et al. [29, 31] reported a total loss of 2.5 days of work for mothers and 0.42–0.8 days for fathers, with a total cost of productivity loss ranging from €234.34 to €257.46 for mothers and from €47.83 to €174.07 for fathers.

Three studies reported the estimated total annual productivity loss costs due to SSTIs at the national level, being €34,623,835.89 in Poland [32], €17,382,154.02 in Argentina [30], and €4,308,232.09 in Hungary [33]. These estimates were based on the annual number of cases of VZV infection in 2015 in the 3 countries (Table 1). In addition, 3 studies estimated the average costs of childcare by professional caregivers per affected child at €11.95 (Spain) [10], €19.61 (Australia) [29] and €52.12 (United States) [31].

Two cost-effectiveness analyses of vaccination programs against VZV estimated indirect cost saving due to vaccination. Hsu et al. reported an estimated reduction of 98.9% in indirect costs with the implementation of vaccination programs (from €32.00 million to €0.34 million) [25], while in Schuffham et al. the mean indirect cost savings associated with vaccination were €70.60 per child [27].

### Bacterial SSTIs

Three studies from the United States reported indirect costs associated with ABSSSI in children [6, 8, 28]. School days lost due to impetigo ranged from 1.2 to 6.5 days per child. Rice et al. commented that working parents and school-age children were less likely to change their daily activities when receiving topical antibiotic compared to systemic antibiotic treatment (*p* < 0.04) [28]. In an economic simulation model, Lee et al. estimated that the lost productivity associated with each CA-MRSA case in children < 15 years old ranged from €1,814.39 to €8,224.06, based on impetigo, cellulitis and folliculitis [8]. In their study, indirect costs represented 75% of the total costs for CA-MRSA infections. One study compared the hospital indirect costs associated with 2 different treatments of subcutaneous abscess: traditional packing technique vs. minimally invasive (MI) abscess drainage. Median workdays lost decreased from 2 days (interquartile range [IQR] 1–2 days) with the packing treatment to 1 day (IQR 1–1.25 days) with the MI treatment (*p* < 0.001), with a €294.58 reduction in median hospital indirect costs (p < 0.001) per patient and episode [6].

### Pediculosis

A cost-effectiveness analysis from the United States was reviewed [14]. From the caregivers’ perspective, the study compared 3 head lice treatments commonly used in the United States (permethrin 1%, malathion 0.5% and the lice comb) and assumed a treatment duration of 2 weeks. The study estimated school day loss due to head lice infestation to be 1 day in the second week of the treatment, in case of treatment failure, according to the policy recommended by the American Academy of Pediatrics [34]. The total indirect costs per child estimated as the total formal care at home in 1 day was €68.57, plus €21.41 due to time lost by parents in purchasing treatment.

## Discussion

In this systematic review we collected and summarized all the current data on the indirect costs associated with SSTIs in children between 1 and 12 years of age, encompassing 3 highly contagious skin diseases: ABSSSIs, chickenpox and head lice (Table 1). Although some reviews of the economic burden of chickenpox have been published [35–37], this is the first systematic review of studies specifically addressing the indirect cost of SSTIs caused by bacterial, viral, and parasitic pathogens in children. The current review identified studies from various countries (in North America, South America, Europe, Asia and Oceania) with diverse healthcare systems and socioeconomic status.

### Varicella-zoster virus

Regarding VZV infection, the 9 studies reviewed were conducted in 9 countries with a variety of healthcare systems and standards of care and living, making it difficult to compare or to analyze the underlying reasons for the cost differences among countries. In addition, the indirect costs identified in this review varied considerably due to the difference in components; thus, the indirect costs, defined as the total cost of productivity loss reported for uncomplicated chickenpox, were around €200 in the United States, Australia and Poland [29, 31, 32], around €100 in Hungary, Argentina and New Zealand [27, 33], less than €100 in Spain and Taiwan [10, 25], and as low as €1.16 in Brazil [26]. The number of lost workdays for caregivers was heterogeneous, ranging from 0.27 days in Brazil to 7.8 days in Argentina [26, 30]. Despite this variation, SSTIs can cause a substantial number of lost workdays for the parents of sick children. In addition, 4 studies reported higher indirect costs for the caregivers of patients who required hospitalization compared to patients that were treated at home [26, 27, 32, 33]. Conversely, Giglio et al. reported fewer workdays lost and therefore less productivity loss for inpatients vs. outpatients [30]. This difference arose because the authors considered the time spent in the hospital but not the additional time spent recovering at home. Interestingly, 2 studies reported higher productivity loss for working mothers. These differences were attributable to mothers missing more days of work due to childcare than fathers, in addition to the gender pay gap [29, 31]. However, both studies were published more than 20 years ago and would need to be updated using current information.

The total annual indirect costs were substantially different between Poland, Argentina, and Hungary (€34,623,835.89; €17,382,154.02; and €4,308,232.09, respectively). With a similar incidence of VZV and cost of lost workdays, these differences were mainly due to the differences in the number of VZV cases reported in 2015 in the 3 countries (Table 1) [30, 32, 33].

The introduction of vaccines reduces VZV burden of disease by about 80–85% [38] and has a number of benefits, such as reducing disease-related complications, healthcare costs, and worktime lost. The cost-benefit analyses showed substantial overall savings in indirect costs resulting from reduced worktime loss associated with vaccination [25, 27]. In many countries, VZV vaccination is either not mandatory or is gradually being introduced into the national immunization program, therefore current data on disease burden and vaccine implementation and coverage is limited [37].

### Bacterial SSTIs

Although ABSSSI represents a significant economic burden for health systems and individual patients worldwide, we found only 3 studies from the United States that reported the indirect costs of SSTIs produced by bacterial infection in the pediatric population [6, 8, 28].

The article published by Lee et al. [8] was the only study that included productivity loss due to absenteeism and mortality in the estimation of total cost of productivity loss, in this case, due to impetigo, cellulitis and folliculitis produced by CA-MRSA infection. The authors estimated productivity losses associated with children under 15 years to be between €1,814.39 and €8,224.06. In the economic analysis, they found that societal costs, including indirect costs, were 4 to 7 times higher than healthcare medical costs, as the vast majority came from productivity loss (i.e. individuals or caregivers missing work plus lost productivity from infection-related deaths). A non-surviving child represents a greater productivity loss than an adult if we consider that they would be expected to have a whole productive life ahead of them, while an adult has already contributed a part of their productive life. In a systematic review, median childhood impetigo prevalence was estimated at 12.3% (IQR 4.2-19.4%) [39]. In Spain, with nearly 5.9 million children [40], this would represent a total indirect cost ranging from €1,270,073 to €5,756,842 per year due to impetigo.

Another study presented a cost-effectiveness analysis of the treatment of impetigo with erythromycin or mupirocin. The study concluded that treatment with mupirocin (topical) reduced indirect costs due to a decreased number of school days and workdays lost compared with treatment with erythromycin (oral) [28].

Finally, the indirect costs reported by Wright et al. in the treatment of subcutaneous abscess in children were higher with traditional packing than with MI drainage, because the latter reduced hospital length of stay [6]. However, the study does not present the full methodology used to obtain direct costs, thus it is difficult to interpret their results. Annual recurrence is frequent in patients with SSTI caused by CA-MRSA. The use of mupirocin (MUP) based decolonization is recommended for patients with recurrent SSTI or in settings of ongoing transmission [41]. However, following its widespread use, the emergence of MUP resistance is increasing among MRSA isolates worldwide, and may represent a substantial health burden [42].

### Pediculosis

Although *pediculosis capitis* is the most frequent ectoparasitic disease worldwide, and its treatment is costly, indirect costs associated with head lice were found in only 1 cost-effectiveness study, which compared 3 *pediculosis capitis* treatments [14]. While head lice infestation is not a public health hazard, it merits study because of its increasing incidence and the associated healthcare costs. The cost-effectiveness analysis published by Gur et al., reported that 73% of the total variability of the model was attributed to the number of lost school days and subsequent lost caregiver workdays. The authors emphasized that a head lice policy in schools has an important effect on the cost and effectiveness of the different treatments [14]. Nevertheless, the findings of this study are based on an assumed loss of school days and may not reflect the actual burden of head lice. A 1997 report estimated that approximately 6 to 12 million infestations occur each year in the United States [43], representing between €411.42 million and €822.84 million in indirect costs.

One important limitation of this study is the scarcity of cost-of-illness studies in SSTIs in the pediatric population, and of those that exist, many are based on data from clinical practice dating from 2005 or earlier. This could underestimate the true extent of the disease burden. In addition, the indirect costs included in this systematic review derived from a heterogeneous group of studies with different methodologies. We included studies related to different diseases, which may not therefore be directly comparable. In this regard, the studies included were conducted in different countries in which indirect costs are estimated according to the country-specific features of the labor market, which makes it difficult to directly compare costs across studies. Furthermore, the methodology used in some studies was not clearly described, or the results were presented as aggregated costs, and thus could not be analyzed in detail. Hence, new studies that analyze the cost-of-illness of SSTIs in pediatric patients should be designed and should present results not only as aggregated costs. This would enable the implementation of tailored strategies and policies. Additionally, these studies should include data from as many different SSTIs as possible so they can be evaluated as a whole.

Aside from the scarce data on indirect costs of SSTIs in the pediatric population, the QoL of these patients is also underreported. These skin disorders may cause several disturbances, stigmatization, and psychosocial consequences that affect the lives of both the patients and their families. Therefore, it is of utmost importance to assess to a greater extent the impact of these diseases on the overall QoL in pediatric patients by designing studies evaluating QoL outcome measurements [44, 45]. This burden is especially relevant in children with highly contagious skin diseases, outbreaks and relapses. In addition, the availability of scales measuring QoL in ABSSSIs is not well known and accurate instruments should be developed. Thus, further investigation is needed to obtain a comprehensive picture of the real burden of these diseases in the pediatric population.

## Conclusions

In conclusion, there is no doubt that productivity losses attributable to SSTIs in children are extremely important. The economic burden of SSTIs, though highly relevant, is underestimated due to the lack of studies, particularly in bacterial SSTIs. Although many cost-of-illness studies acknowledge the importance of indirect costs and benefits, they are seldom included in economic assessments. New, improved cost studies using a precise, standardized indirect cost estimation methodology will give us a better understanding of the magnitude of the economic disease burden and allow us to determine the efficacy of health policies and develop programs to improve public health.

## Supplementary Information

**Additional file 1 **: **Supplementary Table 1**. PRISMA Checklist.

**Additional file 2 **: **Supplementary Table 2**. Search strategies.

**Additional file 3 **: **Supplementary Table 3**. Result of the quality appraisal.

**Additional file 4 **: **Supplementary Table 4**. Risk of bias in observational studies included in the systematic review.

**Additional file 5 **: **Supplementary Table 5**. Risk of bias assessment of included model-based economic evaluations (based on the ECOBIAS checklist).

## Data Availability

The datasets used and/or analyzed during the current study are available from the corresponding author on reasonable request. The research protocol was registered in Open Science Framework (https://osf.io/wrvap/).

## Abbreviations

ABSSSIs: Acute bacterial skin and skin structure infections
Avg: Average
CA-MRSA: Community-associated (or community-acquired) methicillin-resistant *Staphylococcus aureus*
CCEMG-EPPI: Campbell and cochrane economics methods group
CI: Confidence interval
COI: Cost-of-illness
CRD: Centre for reviews and dissemination
ECOBIAS: Bias in economic evaluation
HZ: Herpes zoster
IQR: Interquartile range
ISPOR: International society of pharmacoeconomics and outcomes research
MI: Minimally invasive
MRSA: Methicillin-resistant *Staphylococcus aureus*
MUP: Mupirocin
NA: Not applicable
NICE: National Institute for Health and Care Excellence
No: Numbers
OECD: Organization for economic cooperation and development
OSF: Open science framework
PRISMA: Preferred reporting items for systematic reviews and meta-analysis
QoL: Quality of life
SSTIs: Skin and soft tissue infections
TP: Traditional packing treatment
VZV: *Varicella-zoster virus*
WoS: Web of science
