# Supplementary material for: Indirect costs associated with skin infectious disease in children: a systematic review
Source: BMC Health Serv Res. 2021 Dec 11;21:1325. doi: 10.1186/s12913-021-07189-3 (PMC8665520; doi:10.1186/s12913-021-07189-3)
Supplement: Supplementary file 2 — Additional file 2 : Supplementary Table 2. Search strategies. [file 12913_2021_7189_MOESM2_ESM.pdf]

Online Resource 2. Search strategies

PubMed	1	(("Skin Diseases, Infectious"[MeSH]) AND "Child"[MeSH]) AND "Cost of Illness"[MeSH]
	2	("Skin Diseases, Infectious"[MeSH] AND "economics"[Subheading]) AND "Child"[MeSH]
	3	(((((("Skin Diseases, Infectious"[MeSH] AND "humans"[MeSH Terms]) OR ("Staphylococcal Skin Infections"[MeSH] AND "humans"[MeSH Terms])) AND "humans"[MeSH Terms]) OR ("Soft Tissue Infections"[MeSH] AND "humans"[MeSH Terms])) AND "humans"[MeSH Terms]) AND "economics"[Subheading]) AND "Child"[MeSH]
	4	(((((("Skin Diseases, Infectious"[MeSH] AND "humans"[MeSH Terms]) OR ("Staphylococcal Skin Infections"[MeSH] AND "humans"[MeSH Terms])) AND "humans"[MeSH Terms]) OR ("Soft Tissue Infections"[MeSH] AND "humans"[MeSH Terms])) AND "humans"[MeSH Terms]) AND "Child"[MeSH]) AND "Cost of Illness"[MeSH]
	5	(((((((("Skin Diseases, Infectious"[MeSH] AND "humans"[MeSH Terms]) OR ("Staphylococcal Skin Infections"[MeSH] AND "humans"[MeSH Terms])) AND "humans"[MeSH Terms]) OR ("Soft Tissue Infections"[MeSH] AND "humans"[MeSH Terms])) AND "humans"[MeSH Terms]) AND "Child"[MeSH]))) AND "Health Expenditures"[MeSH]
	6	(((((("Skin Diseases, Infectious"[MeSH] AND "humans"[MeSH Terms]) OR ("Staphylococcal Skin Infections"[MeSH] AND "humans"[MeSH Terms])) AND "humans"[MeSH Terms]) OR ("Soft Tissue Infections"[MeSH] AND "humans"[MeSH Terms])) AND "humans"[MeSH Terms]) AND "Health Expenditures"[MeSH]
	7	(("Skin Diseases, Infectious"[MeSH]) AND "Child, Preschool"[MeSH]) AND "Cost of Illness"[MeSH]
	8	("Skin Diseases, Infectious"[MeSH] AND "economics"[Subheading]) AND "Child, Preschool"[MeSH])
	9	(((((("Skin Diseases, Infectious"[MeSH] AND "humans"[MeSH Terms]) OR ("Staphylococcal Skin Infections"[MeSH] AND "humans"[MeSH Terms])) AND "humans"[MeSH Terms]) OR ("Soft Tissue Infections"[MeSH] AND "humans"[MeSH Terms])) AND "humans"[MeSH Terms]) AND "economics"[Subheading]) AND "Child, Preschool"[MeSH]
	10	(((((("Skin Diseases, Infectious"[MeSH] AND "humans"[MeSH Terms]) OR ("Staphylococcal Skin Infections"[MeSH] AND "humans"[MeSH Terms])) AND "humans"[MeSH Terms]) OR ("Soft Tissue Infections"[MeSH] AND "humans"[MeSH Terms])) AND "humans"[MeSH Terms]) AND "Child, Preschool"[MeSH]) AND "Cost of Illness"[MeSH]
	11	(((((((("Skin Diseases, Infectious"[MeSH] AND "humans"[MeSH Terms]) OR ("Staphylococcal Skin Infections"[MeSH] AND "humans"[MeSH Terms])) AND "humans"[MeSH Terms]) OR ("Soft Tissue Infections"[MeSH] AND "humans"[MeSH Terms])) AND "humans"[MeSH Terms]) AND "Child, Preschool"[MeSH]))) AND "Health Expenditures"[MeSH]
	12	"Indirect costs"[All Fields] AND ("skin infections"[All Fields] OR "soft tissue infections"[All Fields])
	13	(((((("Skin Diseases, Infectious"[MeSH] AND "humans"[MeSH Terms]) OR ("Staphylococcal Skin Infections"[MeSH] AND "humans"[MeSH Terms])) AND "humans"[MeSH Terms]) OR ("Soft Tissue Infections"[MeSH] AND "humans"[MeSH Terms])) AND "humans"[MeSH Terms]) AND "indirect costs") AND "Child"[MeSH]
SCOPUS	1	TITLE-ABS-KEY ("skin") AND TITLE-ABS-KEY ("infectious") AND TITLE-ABS-KEY ("disease") AND TITLE-ABS ("child") AND TITLE-ABS-KEY ("cost of illness")
	2	TITLE-ABS-KEY ("skin") AND TITLE-ABS-KEY ("infectious") AND TITLE-ABS-KEY ("disease") AND TITLE-ABS (child* OR pediatric* OR paediatric*) AND TITLE-ABS-KEY (cost* OR economics*)
	3	TITLE-ABS-KEY (("skin" AND "infectious" AND "disease" AND "human") OR ("Staphylococcal Skin Infections" AND "humans") OR ("Soft Tissue Infections" AND "humans")) AND TITLE-ABS (child* OR pediatric* OR paediatric*) AND TITLE-ABS-KEY (cost* OR economics*)
	4	TITLE-ABS-KEY (("skin" AND "infectious" AND "disease" AND "human") OR ("Staphylococcal Skin Infections" AND "humans") OR ("Soft Tissue Infections" AND "humans")) AND TITLE-ABS (child* OR pediatric* OR paediatric*) AND TITLE-ABS-KEY (cost* OR economics*)
	5	TITLE-ABS-KEY (("skin" AND "infectious" AND "disease" AND "human") OR ("Staphylococcal Skin Infections" AND "humans") OR ("Soft Tissue Infections" AND "humans")) AND TITLE-ABS (child* OR pediatric* OR paediatric*) AND TITLE-ABS-KEY (health expenditure* OR expenditure*)
	6	TITLE-ABS-KEY (("skin" AND "infectious" AND "disease" AND "human") OR ("Staphylococcal Skin Infections" AND "humans") OR ("Soft Tissue Infections" AND "humans")) AND TITLE-ABS-KEY (health expenditure* OR expenditure*)
	7	TITLE-ABS-KEY (("skin" AND "infectious" AND "disease" AND "human") OR ("Staphylococcal Skin Infections" AND "humans") OR ("Soft Tissue Infections" AND "humans")) AND TITLE-ABS-KEY (child* OR pediatric* OR paediatric* OR preschool*) AND TITLE-ABS-KEY (burden* AND illness*)
	8	TITLE-ABS-KEY ("skin" AND "infectious" AND "disease") AND TITLE-ABS (child* OR pediatric* OR paediatric* OR preschool*) AND TITLE-ABS-KEY (cost* OR economics*)
	9	TITLE-ABS-KEY (("skin" AND "infectious" AND "disease" AND "human") OR ("Staphylococcal Skin Infections" AND "humans") OR ("Soft Tissue Infections" AND "humans")) AND TITLE-ABS (child* OR pediatric* OR paediatric*) AND TITLE-ABS-KEY (economics*) AND TITLE-ABS (child* OR pediatric* OR paediatric* OR preschool*)
	10	TITLE-ABS-KEY (("skin" AND "infectious" AND "disease" AND "human") OR ("Staphylococcal Skin Infections" AND "humans") OR ("Soft Tissue Infections" AND "humans")) AND TITLE-ABS-KEY (child* OR pediatric* OR paediatric* OR preschool*) AND TITLE-ABS-KEY (economics* OR cost*)
	11	TITLE-ABS-KEY (("skin" AND "infectious" AND "disease" AND "human") OR ("Staphylococcal Skin Infections" AND "humans") OR ("Soft Tissue Infections" AND "humans")) AND TITLE-ABS-KEY (child* OR pediatric* OR paediatric* OR preschool*) AND TITLE-ABS-KEY (health expenditure* OR expenditure*)
	12	ALL ("Indirect costs") AND ALL (("skin infections") OR ("soft tissue infections"))

	13	TITLE-ABS-KEY (("skin" AND "infectious" AND "disease" AND "human") OR ("Staphylococcal Skin Infections" AND "humans") OR ("Soft Tissue Infections" AND "humans")) AND TITLE-ABS (child* OR pediatric* OR paediatric*) AND TITLE-ABS-KEY ("indirect costs")
WOS	1	TS=("skin" AND "infectious" AND "disease") AND TS=(child* OR pediatric* OR paediatric*) AND TS=("cost of illness")
	2	TS=("skin" AND "infectious" AND "disease") AND TS=(child* OR pediatric* OR paediatric*) AND TS=(cost* OR economics*)
	3	TS=(("skin" AND "infectious" AND "disease" AND "human") OR ("Staphylococcal Skin Infections" AND "humans") OR ("Soft Tissue Infections" AND "humans")) AND TS=(child* OR pediatric* OR paediatric*) AND TS=(cost* OR economics*)
	4	TS=(("skin" AND "infectious" AND "disease" AND "human") OR ("Staphylococcal Skin Infections" AND "humans") OR ("Soft Tissue Infections" AND "humans")) AND TS=(child* OR pediatric* OR paediatric*) AND TS=(cost* OR economics*)
	5	TS=(("skin" AND "infectious" AND "disease" AND "human") OR ("Staphylococcal Skin Infections" AND "humans") OR ("Soft Tissue Infections" AND "humans")) AND TS=(child* OR pediatric* OR paediatric*) AND TS=(health expenditure* OR expenditure*)
	6	TS=(("skin" AND "infectious" AND "disease" AND "human") OR ("Staphylococcal Skin Infections" AND "humans") OR ("Soft Tissue Infections" AND "humans")) AND TS=(health expenditure* OR expenditure*)
	7	TS=(("skin" AND "infectious" AND "disease" AND "human") OR ("Staphylococcal Skin Infections" AND "humans") OR ("Soft Tissue Infections" AND "humans")) AND TS=(child* OR pediatric* OR paediatric* OR preschool*) AND TS=(burden* AND illness*)
	8	TS=("skin" AND "infectious" AND "disease") AND TS=(child* OR pediatric* OR paediatric* OR preschool*) AND TS=(cost* OR economics*)
	9	TS=(("skin" AND "infectious" AND "disease" AND "human") OR ("Staphylococcal Skin Infections" AND "humans") OR ("Soft Tissue Infections" AND "humans")) AND TS=(economics*) AND TS=(child* OR pediatric* OR paediatric* OR preschool*)
	10	TS=(("skin" AND "infectious" AND "disease" AND "human") OR ("Staphylococcal Skin Infections" AND "humans") OR ("Soft Tissue Infections" AND "humans")) AND TS=(child* OR pediatric* OR paediatric* OR preschool*) AND TS=(economics* OR cost*)
	11	TS=(("skin" AND "infectious" AND "disease" AND "human") OR ("Staphylococcal Skin Infections" AND "humans") OR ("Soft Tissue Infections" AND "humans")) AND TS=(child* OR pediatric* OR paediatric* OR preschool*) AND TS=(health expenditure* OR expenditure*)
	12	ALL=("Indirect costs") AND ALL=(("skin infections") OR ("soft tissue infections"))
	13	TS=(("skin" AND "infectious" AND "disease" AND "human") OR ("Staphylococcal Skin Infections" AND "humans") OR ("Soft Tissue Infections" AND "humans")) AND TS=(child* OR pediatric* OR paediatric*) AND TS=("indirect costs")
