# Supplementary material for: Indirect costs associated with skin infectious disease in children: a systematic review
Source: BMC Health Serv Res. 2021 Dec 11;21:1325. doi: 10.1186/s12913-021-07189-3 (PMC8665520; doi:10.1186/s12913-021-07189-3)
Supplement: Supplementary file 3 — Additional file 3 : Supplementary Table 3. Result of the quality appraisal. [file 12913_2021_7189_MOESM3_ESM.pdf]

Online Resource 3. Result of the quality appraisal

	Was the study question well specified?	Were quantification methods appropriate and well-executed?	Were healthcare resources valued appropriately?	Was the inclusion of indirect costs appropriate?	Was a range of estimates presented?	Was a sensitivity analysis performed?	Was uncertainty around the estimates and its implications adequately discussed?	Were important limitations discussed regarding the cost components, data, assumptions, and methods?	Will the results help to understand indirect costs of SSTIs?
Díez-Domingo [10]	1	1	1	1	2	2	2	2	1
Ferson [29]	1	1	3	3	1	2	2	2	1
Giglio [30]	1	1	1	1	1	1	1	1	1
Gur [14]	1	1	1	1	1	1	1	1	1
Hsu [25]	1	1	1	1	1	1	1	1	1
Lee [8]	1	1	1	2	1	1	1	1	1
Lieu [31]	1	1	3	1	1	2	2	1	1
Meszner [33]	1	1	1	1	1	1	1	1	1
Rice [28]	1	1	1	1	1	2	2	1	1
Scuffham [27]	1	1	1	1	1	1	1	1	1
Valentim [26]	1	1	1	1	1	1	1	1	1
Wright [6]	1	3	3	1	1	2	2	1	1
Wysocki [32]	1	1	1	3	1	2	2	1	1

Legend: Yes 1 No 2 Can't Say 3 Not Applicable 0
