# Supplementary material for: Indirect costs associated with skin infectious disease in children: a systematic review
Source: BMC Health Serv Res. 2021 Dec 11;21:1325. doi: 10.1186/s12913-021-07189-3 (PMC8665520; doi:10.1186/s12913-021-07189-3)
Supplement: Supplementary file 4 — Additional file 4 : Supplementary Table 4. Risk of bias in observational studies included in the systematic review. [file 12913_2021_7189_MOESM4_ESM.pdf]

**Online Resource 4.** Risk of bias in observational studies included in the systematic review [23]

| **Quality Assessment** | **Díez-Domingo [10]** | **Ferson [29]** | **Giglio [30]** | **Lieu [31]** | **Meszner [33]** | **Rice [28]** | **Wright [6]** | **Wysocki [32]** |
|---|---|---|---|---|---|---|---|---|
| 1. Case series collected in more than one center, i.e. multi-center study | 0 | 0 | 1 | 0 | 1 | 0 | 0 | 1 |
| 2. Is the hypothesis/aim/objective of the study clearly described? | 1 | 1 | 1 | 1 | 1 | 1 | 1 | 1 |
| 3. Are the inclusion and exclusion criteria (case definition) clearly reported? | 1 | 1 | 1 | 0 | 1 | 1 | 1 | 1 |
| 4. Is there a clear definition of the outcomes reported? | 1 | 1 | 1 | 1 | 1 | 1 | 1 | 1 |
| 5. Were data collected prospectively? | 0 | 0 | 0 | 0 | 0 | 1 | 0 | 0 |
| 6. Are the main findings of the study clearly described? | 1 | 1 | 1 | 1 | 1 | 1 | 1 | 1 |
| 7. Are outcomes stratified? (e.g., by disease stage, abnormal test results, patient characteristics) | 1 | 1 | 1 | 1 | 1 | 1 | 1 | 1 |
| **Criteria each study fulfilled (%)** | **71.4%** | **71.4%** | **85.7%** | **57.1%** | **85.7%** | **85.7%** | **71.4%** | **85.7%** |

Yes=1 No=0
