# Supplementary material for: Indirect costs associated with skin infectious disease in children: a systematic review
Source: BMC Health Serv Res. 2021 Dec 11;21:1325. doi: 10.1186/s12913-021-07189-3 (PMC8665520; doi:10.1186/s12913-021-07189-3)
Supplement: Supplementary file 5 — Additional file 5 : Supplementary Table 5. Risk of bias assessment of included model-based economic evaluations (based on the ECOBIAS checklist). [file 12913_2021_7189_MOESM5_ESM.pdf]

Online Resource 5. Risk of bias assessment of included model-based economic evaluations (based on the ECOBIAS checklist) [24]

Type of bias	Gur [14]	Hsu [25]	Lee [8]	Scuffham [27]	Valentim [26]	Studies that fulfilled the respective criterion (%)
**PART A. Overall checklist for bias in economic evaluation**						
Narrow perspective bias		1	1	1	1	80%
Inefficient comparator bias	1	1		1		60%
Cost measurement omission bias	1	1	1	1	1	100%
Intermittent data collection bias	NA	NA	NA	NA	NA	0%
Invalid valuation bias	1	1	1	1	1	100%
Ordinal ICER bias	1				1	40%
Double-counting bias						0%
Inappropriate discounting bias		1		1	1	60%
Limited sensitivity analysis bias§	1	1		1	1	80%
Sponsor bias	NA	NA	NA	NA	NA	0%
Reporting and dissemination bias	NA	NA	NA	NA	NA	0%
**PART B. Model-specific aspects of bias in economic evaluation**						
I Bias related to structure						
Structural assumptions bias	1	1	1	1	1	100%
No treatment comparator bias*	1	1	1	1	1	100%
Wrong model bias	1	1	1	1	1	100%
Limited time horizon bias	1			1		40%
II Bias related to data						
Bias related to data identification	1	1	1	1	1	100%
Bias related to baseline data		1	1	1	1	80%
Bias related to treatment effects		1		1		40%
Bias related to quality-of-life weights (utilities)	NA	NA	NA	NA	NA	0%
Non-transparent data incorporation bias	1	1	1	1	1	100%
Limited scope bias§	1	1	1	1	1	100%
III Bias related to consistency						
Bias related to internal consistency	NA	NA	NA	NA	NA	0%
**Criteria each study fulfilled (%)**	**70.6%**	**82.4%**	**58.8%**	**88.2%**	**76.5%**	

*These biases are overlapping regarding their content. §These biases are overlapping regarding their content. 1: Criterion fulfilled, ECOBIAS: Bias in Economic Evaluation, NA: not applicable.
